# Recent Advances in Non-Targeted Screening of Compounds in Plastic-Based/Paper-Based Food Contact Materials

**DOI:** 10.3390/foods12224135

**Published:** 2023-11-15

**Authors:** Ya Chen, Hongyan Li, Haizhi Huang, Biao Zhang, Zihong Ye, Xiaoping Yu, Xuping Shentu

**Affiliations:** 1 College of Life Science, China Jiliang University, Hangzhou 310018, China; yachen_alisa@yeah.net; 2 Zhejiang Institute of Product Quality and Safety Science, Hangzhou 310018, China; yourlhy@163.com; 3 Zhejiang Provincial Key Laboratory of Biometrology and Inspection & Quarantine, China Jiliang University, Hangzhou 310018, China; zhangbiao9129@163.com (B.Z.); zhye@cjlu.edu.cn (Z.Y.); yxp@cjlu.edu.cn (X.Y.)

**Keywords:** food contact materials, compound, non-targeted screening, high-resolution mass spectrometry, research progress

## Abstract

Ensuring the safety of food contact materials has become a pressing concern in recent times. However, detecting hazardous compounds in such materials can be a complex task, and traditional screening methods may not be sufficient. Non-targeted screening technologies can provide comprehensive information on all detectable compounds, thereby supporting the identification, detection, and risk assessment of food contact materials. Nonetheless, the non-targeted screening of food contact materials remains a challenging issue. This paper presents a detailed review of non-targeted screening technologies relying on high-resolution mass spectrometry for plastic-based and paper-based food contact materials over the past five years. Methods of extracting, separating, concentrating, and enriching compounds, as well as migration experiments related to non-targeted screening, are examined in detail. Furthermore, instruments and devices of high-resolution mass spectrometry used in non-targeted screening technologies for food contact materials are discussed and summarized. The research findings aim to provide a theoretical basis and practical reference for the risk management of food contact materials and the development of relevant regulations and standards.

## 1. Introduction

With the evolution of society, there has been a growing emphasis on ensuring food safety. Part of this emphasis is on the safety of food contact materials (FCMs), which play a crucial role in the food industry. FCMs refer to materials that come into contact with food during various stages of processing, transportation, marketing, and storage, whether directly or indirectly. As such, they have garnered increased attention in recent years. In modern times, plastic, paper, and other comparable materials are frequently applied as FCMs. Typical examples of such materials include plastic bags, packaging boxes made of plastic or paper, and composite films/bags utilized for food. Nonetheless, owing to the distinct qualities of these materials and the properties of the food with which they interact, the chemical constituents present in FCMs may migrate into the food throughout processing, transportation, and storage, presenting a potential danger to human health [1,2,3,4,5].

Currently, the safety hazards associated with FCMs primarily stem from the additives or processing aids used during production and processing, as well as the byproducts that result from storage and sales, degradation products, and residual impurities in raw materials used for manufacturing [6,7,8,9,10]. It is possible for these additives, processing aids, and derivatives added during the manufacture and storage of FCMs to migrate to food through various mechanisms, leading to potential food safety hazards and posing a health risk to humans [11,12]. Given the complex nature and unknown quantity of chemical substances in FCMs, many factors defy prediction, and its migration to food during use is difficult to detect and quantify, thus creating significant challenges for its identification, quantification detection, and safety assessment. Recognizing the vital role of scientific research and practical applications, it is imperative that we undertake in-depth studies on the migration of compounds in FCMs. Furthermore, the ability to use compounds in FCMs to perform safety risk assessment and the management of FCMs are crucial to ensuring food safety and protecting public health.

**Figure 1:**
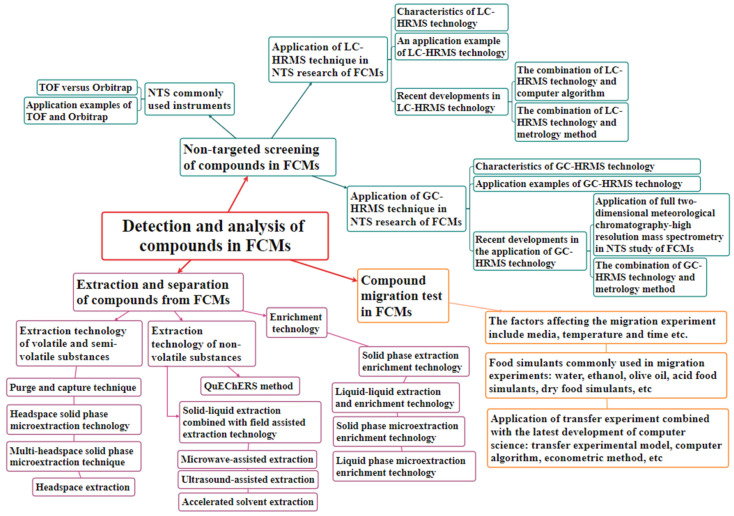
Analysis of compounds in FCMs.

As shown in Figure 1, this review paper discusses recent advancements in the adoption of non-targeted screening (NTS) techniques based on high-resolution mass spectrometry (HRMS) for plastic-based and paper-based FCMs. Methods and technologies used for compound extraction, separation, concentration, and enrichment, as well as migration experiments related to compound NTS in FCMs, are systematically summarized. This paper highlights the importance of liquid chromatography (LC)/gas chromatography (GC)-HRMS instrument technology, metrology methods, and computer programs related to NTS technologies for FCMs. Overall, the purpose of this paper is to provide guidelines for enhancing the ability to detect and analyze compounds in FCMs, thus providing a theoretical framework and practical advice for research into FCM detection, risk management, and the development of related laws and regulations.

## 2. Separation, Extraction, and Migration Experiments of Chemical Substances in Plastic/Paper-Based FCMs

### 2.1. Separation and Extraction of Chemical Compounds in Plastic/Paper-Based FCMs

Plastic/paper-based FCMs are widely used in the market due to their abundance and low cost [13,14,15]. The synthetic processing process of FCMs involves the addition of various additives or processing aids, which contribute to the formation of the resulting materials equipped with specific properties. Due to this, the chemical compounds present in FCMs are diverse, complex, and difficult to predict [16,17]. In the course of experimentation, the extraction and separation of FCMs are usually carried out by carefully selecting the most suitable extraction and separation technology based on both physical and chemical properties of the target compounds. For example, this includes considerations such as volatile, semi-volatile, and non-volatile chemicals. In order to enhance the concentration of the intended target and minimize potential interferences in the experimental matrix, it is particularly significant to choose appropriate enrichment and concentration techniques that are tailored to the specific requirements of the experiment. By making a well-informed and realistic decision, it is possible to achieve optimal experimental results that satisfy the desired objectives.

Table 1 provides an overview of commonly used extraction, separation, and concentration techniques for the detection of plastic- and paper-based FCMs, together with an assessment of their respective advantages and disadvantages. Notably, these techniques are illustrated by various examples. To extract volatile and semi-volatile compounds from FCMs, headspace extraction (HS) and its derivative methods are primarily adopted. On the other hand, solid–liquid extraction combined with field-assisted extraction technology is utilized for extracting non-volatile compounds. The QuEChERS method is capable of meeting the impurity purification requirements for FCM detection. Enrichment and concentration technology is predominantly employed in FCMs to boost the detection of chemical compounds with low concentrations. Recent advancements in this field include thin-film microextraction, covalent organic polymers, computer online systems, among others. 

### 2.2. Migration Experiment

The Chinese food safety standards GB5009.156-2016 [61] and GB31604.1-2015 [62], along with Commission Regulation EU No. 10/2011 [63] have established specific guidelines regarding additives, food simulants, and experimental conditions for FCMs. These regulations also require migration tests to be conducted, which simulate the potential worst-case scenario for the use of FCMs [51,64]. Chemical agents known as food simulants are utilized to simulate food in scientific experiments. Owing to the influence of various food components and properties on the migration experiment results, the application of food simulants that share similarities with certain foods enables the more precise reflection and demonstration of component migration in FCMs. This approach mitigates individual variances and produces experimental outcomes that are more representative of real-world scenarios [65].

Table 2 provides a summary of various migration experiments on plastic/paper-based FCMs [66,67,68,69,70,71,72,73,74,75,76]. The migration behavior of chemical substances in plastic/paper FCMs is determined by a variety of factors such as contact media, temperature, and time, resulting in varying levels of migration under different conditions, affecting the accuracy of final migration data and product compliance assessment [77,78,79,80]. Plastic/paper-based FCMs are usually tested using food simulants like water, ethanol, olive oil, acid, Tenax^®^, and Porapak^®^ [66,81,82,83,84].

Ja’en et al. [68] employed a modified form of polyphenylates as a food simulant to investigate the migration of MOAH from primary paper box packaging into dry food products. A multivariate analysis algorithm was utilized to establish correlations and groupings between the migration patterns of model substances. Furthermore, the worst-case migration scenario of model substances was forecasted using partial least squares regression. The researchers discovered that the migration patterns of model substances were strongly linked to the volatility of the compounds, as well as the composition and characteristics of the food products, the duration of the migration process, and the temperature at which the migration occurred. Consequently, distinct migration behaviors between the most volatile and heaviest model substances could be observed. Lerch et al. [72] conducted a study to examine the migration of three PFAS subclasses from six FCMs to 50% and 20% ethanol, with the aim of fully assessing the impact of PFAS migration from paper FCMs on consumers. Their findings revealed that PFCAs and FTOHs manifested higher levels of migration to 50% ethanol, but surprisingly, FTOHs did not migrate to 20% ethanol, which could have significant implications for public health. This study estimated that children’s diets could contain PFAS levels that exceed the safety threshold recommended by the European Food Safety Authority, which could put their health at risk.

Selecting appropriate food simulators for migration experiments can recreate the contact process between food and FCMs in real-world applications, resulting in the proper and objective assessment of the release or migration of target substances in FCMs and ultimately determining the compliance or safety of FCMs.

### 2.3. Recent Developments and Applications of Migration Experiments

The convergence of computer science and various academic fields has led to the emergence of novel methodologies, while numerous experimental models, computer algorithms, and econometric techniques have been leveraged to evaluate the transferability of research findings [85,86].

Ciffroy et al. [87] developed a simple mechanistic model describing the migration of chemicals from FCMs to food and combined predicted diffusion coefficients and FCM-food distribution coefficients through combining it with quantitative property–property relationships. The performance of the operational model in predicting chemical concentrations in food in contact with plastic monolayer FCMs was evaluated through comparison with experimental migration values reported in the literature. The predicted values agreed well with the experimental values, and the tested model could be used to provide insights into the amount and type of toxicological data required for the safety assessment of FCM compounds, and exhibited a reasonable safety factor. Wang et al. [88] designed a nonlinear machine learning model that takes into account chemical properties, material type, and food type to predict chemical migration from packaging to food and evaluated the predictive performance of nine nonlinear algorithms, taking advantage of integrated models of multiple algorithms to provide more advanced performance. Predictive models after development were subsequently applied to assess the migration potential of food contact chemicals with concerns of high toxicity. This process is expected to facilitate and expedite the evaluation of the transfer of such chemicals from packaging materials to food items, ultimately contributing to safer food supply and management.

The application of the migration program not only accelerates the experimental process, but also reduces its difficulty. It improves the feasibility of experiments and provides a good starting point for further research. It also connects well with other experiments, presenting great development potential.

## 3. NTS MS Analysis of Compounds in Plastic/Paper-Based FCMs

The development and research of FCMs involve a complex analytical calculation process, where traditional targeted screening falls short in adequately identifying trace compounds. To effectively identify trace compounds with low migration levels, complex origins, and unknown information, the NTS method was established using highly sensitive HRMS instruments. The most commonly used NTS method for FCM research is MS detection based on GC or LC coupling, with HRMS being the primary analytical technique [89]. Currently, MS techniques such as time-of-flight (TOF)-MS and Orbitrap-MS are commonly employed by NTS to identify compounds in plastic/paper-based FCMs.

### 3.1. TOF Compares Orbitrap and Its Application Instance

Today, the commonly utilized HRMS instruments for NTS mainly comprise TOF and Orbitrap. The TOF instrument works by accelerating different ions to the same kinetic energy through an electric field, and then utilizing time-of-flight technology to determine the ion speed by the mass–charge ratio. This instrument ensures high resolution and is ideal for NTS research. However, it suffers from a low sensitivity to mixed samples and a weak quantitative ability due to its limited ion trap capacity. Thermo’s Orbitrap is a unique technology that utilizes orbital ion traps. With its high resolution, high accuracy, and low maintenance requirements, Orbitrap stands out from other technologies. While TOF benefits from faster scanning speeds and the capability to work independently, Orbitrap shines in its advantages of resolution, accuracy, sensitivity, stability, and linear range, enabling it to be an optimal choice for researchers.

Yusa et al. [90] developed a novel automated approach to analyze unknown components within the context of recycled low-density polyethylene (LDPE) plastics using the high-resolution mass spectrometer MS^3^ mode of LC-Orbitrap Tribrid-HRMS, with the aid of Compound Discoverer data processing software. A total of 28 unknown compounds were preliminarily identified through this process, many of which belonged to plastic additives. Using LC-q-Orbitrap, Blanc-Zubiaguirre et al. [91] successfully detected the migration of both target and non-target compounds in paper and paperboard materials. They discovered 97 chemicals included in European regulations, such as photoinitiators, phthalates, fungicides, antioxidants, and more. In addition to these compounds, several other substances that were not listed in regulations were also found. A novel approach by Sapozhnikova et al. [92] based on the combination of LC-HRMS and the calculation of octanol-water partition coefficients determines the retention time for the migration testing of common paper FCMs such as pizza boxes, pizza box linings, and slaughtering paper. To further reduce false positive results, an orthogonal approach using LC retention information is utilized to refine the HRMS data, identifying a total of 153 different migratory compounds.

HRMS is capable of swiftly and accurately determining the precise mass of a compound. With the help of information from the mass spectrum, including molecular ion peaks and product ion peaks, HRMS can precisely identify the structure of unknown compounds, thus effectively identifying chemical compounds migrated from FCMs. When used in conjunction with HRMS, NTS technologies can efficiently gather all necessary migration data and information to provide comprehensive support for risk assessment and management, as well as the legislation of FCMs, making it an invaluable asset.

### 3.2. Application of LC-HRMS Technology in FCM Detection

LC is capable of analyzing a wide range of compounds, including compounds with high boiling points and poor thermal stability, without being affected by volatility or thermal limitations. Moreover, its combination with HRMS enhances the accuracy of compound identification, allowing it to be a valuable tool for qualitative analysis of non-volatile FCM compounds. Its versatility and accuracy have made it a popular technique for analyzing non-volatile FCM compounds (Table 3) [81,93,94,95,96,97,98,99].

Vera et al. [81] adopted UPLC-ion mobility spectroscopy (IMS)-QTOF to investigate PFAS migration in FCMs. The analysis revealed the presence of 11 PFAS compounds in these FCMs, but their concentrations did not exceed the migration limit. Sapozhnikova et al. [95] conducted an extensive investigation into the transmission of chemicals across a range of plastic FCMs, including microtrays, microwave bags, and oven bags used for both microwave and conventional oven cooking. The team employed LC-Orbitrap-MS as a means of identifying and characterizing the chemicals present in FCMs. An effective method for validating NTS was established using isotopically labeled chemicals, and a reliable protocol for identifying chemical migration was formulated. During the initial screening phase, 74 compounds were identified as presenting a potential risk to food safety. Sanchis et al. [96] developed a novel approach for detecting UV ink photoinitiators and PAAs in FCMs by means of LC coupled with LC-Orbitrap-HRMS, resulting in the identification of 10 types of UV ink photoinitiators and 8 types of PAAs in juice milk bags. Their study also included an analysis of 18 different types of packaging samples, where photoinitiators and PAAS found in the UV inks were examined carefully. Recovery rates ranged from 78% to 119%, with RSD values below 20%.

LC-HRMS is a widely utilized instrument for the detection of compounds within FCMs due to its high degree of accuracy in MS, superior separation effect, exceptional ability to separate complex samples, high selectivity, and extremely low detection limit. Meanwhile, the establishment of LC-HRMS databases such as mzcloud or NIST, along with the establishment and continuous refinement of migration databases including Agilent’s Mass Hunter E&L PCDL and Thermo Field’s Thermo Compound Discover E&L HRAM Compound database, have played a great role in the characterization of chemical compounds. 

### 3.3. The Development of LC-HRMS Combined with Computer Algorithms and Metrology Methods

The role of computer tools in NTS research is increasingly significant. The use of applications, mathematical models, and automated processing tools expands the possibilities for conducting NTS analysis of LC-HRMS and broadens the application range of the technique. Diverse metrology methods can fulfill different experimental requirements, resulting in higher efficiency and enhanced data accuracy.

Yusa et al. [90] devised an automated method for surveying unknown compounds in LDPE plastic FCMs through setting up an acqurex™ intelligent data acquisition channel connected to LC-Orbitrap Tribrid-HRMS (MS^3^). With the use of Compound Discoverer data processing, the initial screening and identification of 28 unknown compounds could be realized. Blanco-Zubiaguirre et al. [91] developed an analytical procedure for LC-quadruply-Orbitrap-MS to accurately characterize and verify the migration of both target and non-target compounds onto Tenax^®^ in various types of paper and paperboard materials. This procedure revealed a staggering 97 regulated chemicals and other unlisted compounds present in these materials.

Sapozhnikova et al. [92] performed a study to evaluate the migration of chemicals in common paper FCMs using the LC-HRMS technique. They refined the HRMS data by applying a retention time model based on octanol water partition coefficient values and identified a total of 153 migration chemicals. Xu et al. [100] utilized data from analytical columns comprising four different types of materials, C18, phenylhexyl, pentafluorophenyl, and cyanyl, in conjunction with 178 pure chemical standards, to establish a quantitative structure for retention time in chromatography. This model was confirmed to play a certain role in predicting retention relationships for unidentified compounds in NTS, thereby facilitating the identification process. Fisher et al. [101] conducted a comprehensive analysis of prevalent analytical tools utilized in conjunction with NTS methods in recent years, including but not limited to stoichiometry, compound databases, and molecular networks. They delved into the latest advancements, benefits, and practical applications of these methods, offering guidance to researchers seeking to develop NTS workflows tailored to their specific needs and analyses.

In LC-HRMS research, a diverse set of computational algorithms and metrology techniques have been employed, including compound databases, molecular networks, and stoichiometry, along with the ubiquitous NTS method for FCMs. Advanced computer programs have been developed to facilitate the detection and identification of NTS, taking into account crucial data acquisition and analysis within the context of FCMs. These interconnected topics bring tremendous convenience to carrying out NTS research, making them highly valuable for future investigation.

### 3.4. Application of GC-HRMS Technology in FCM Detection

Presently, the most commonly used GC-HRMS technologies for plastic/paper-based FCMs encompass GC-TOF-HRMS, GC-Orbitrap-MS, and atmospheric pressure gas chromatography (APGC)-HRMS, all of which provide fast separation time and exceptional separation capabilities, particularly suitable for the detection of volatile small-molecule substances (Table 4) [95,102,103,104,105,106,107,108].

Su et al. [103] optimized the technique of APGC-QTOF-MS combined with electron ionization MS and commercial library retrieval, thereby proposing effective compound analysis strategies based on different levels of confidence, which were subsequently successfully applied to food-contact-grade polypropylene samples. Ja´en et al. [104] succeeded in screening and identifying the most reliable markers for aromatic mineral oil in FCMs through a combination of allophycocyanin-QTOF-MS and APGC-QTOF-MS techniques. In addition, specific analysis revealed MOAH in a range of recycled materials, including polypropylene-based products, recycled paperboard, and other similar materials. A total of 27 distinct markers were found in mineral oil samples, with 22 identified in recovered polyethylene terephthalate (rPET) and eight in recycled cardboard products. Sapozhnikova [105] detected migrating compounds in paper FCMs through GC-Orbitrap-MS and developed a novel approach for automatic data processing. A total of 35 migratory compounds were identified, consisting of natural compounds, ethyl fatty acid esters, phthalates, and additives, of which 23 exhibited significant changes in their migration levels.

The primary use of GC-HRMS technologies is for detecting volatile targets, which provides precise mass measurements and comprehensive full-scan spectroscopy. These capabilities enable a broad range of qualitative analyses of FCMs containing VOCs to be conducted.

### 3.5. Development of Full Two-Dimensional Meteorological Chromatography (GC × GC)-HRMS for NTS Detection of FCMs

GC × GC-HRMS is a GC method that comprises two independently operated columns, in series, utilizing differing separation mechanisms. The modulator serves as both capture and transfer mechanisms, allowing fractions separated by the first column to be focused and then directed to the second column for further separation by pulse mode. Following separation, the signal strength, representing the intensity of each peak, is processed using the data processing system, which results in a three-dimensional chromatogram, with the retention time of column 1 as the first horizontal coordinate, the retention time of column 2 as the second horizontal coordinate, and signal strength as the longitudinal coordinate. 

Carralero et al. [109] employed the technique of GC × GC-TOF-MS for the analysis of VOCs and SVOCs present in food simulants, which had migrated from polypropylene FCMs to water, 3% acetic acid, 10% ethanol, and other similar environments. A total of 107 different volatile analytes were detected. Wu et al. [110] developed a novel method for detecting SVOCs in mechanical rPET for FCMs. On the basis of a combination of GC×GC-TOF-MS analysis and stoichiometric methods, this method ensures multiple stoichiometric calculations on the data to determine differences in concentrations. Additionally, potential factors that could influence the results were explored, such as the degree of polymerization and molecular weight. Li et al. [111] utilized HS-SPME in combination with GC × GC-QTOF-MS technology to examine VOCs present in food contact cardboard. The specific analysis yielded a total of 331, 191, 154, and 295 VOCs belonging to six different chemical categories.

The advancement of GC×GC enhances the peak capacity and resolution of GC, elevates its sensitivity and signal-to-noise ratio, and resolves the issues of GC-overlapping peaks, co-distillation peaks, and the incompleteness of separation. The integration of GC × GC with HRMS has simplified the processes of qualitative and quantitative analyses, with greater accuracy and more convenience. Moreover, it has opened up new avenues of research for the investigation of NTS phenomena in FCMs, as it has been reflected in various studies in recent years.

### 3.6. Development of Analytical Methods for GC-HRMS

With the rapid advancement of GC-HRMS, a reliable instrument foundation and abundant data have been provided to support the analysis of compounds present in FCMs. To thoroughly examine the detected compounds, researchers have adopted flexible stoichiometric methods to delve deeper into the findings.

Hao et al. [112] employed the combination of direct injection/GC × GC-QTOF-MS and various stoichiometric NTS methods to successfully identify a total of 105 kinds of SVOCs in both regenerative and primary PET. A total of 267 different SVOCs were initially uncovered. Utilizing orthogonal partial least squares discriminant analysis combined with principal component analysis and complemented with non-parametric tests, it was discovered that certain labels might originate from food, drugs, cosmetics, and other sources. Many of these substances were proved to be highly toxic chemicals, necessitating closed-loop recycling and segregation for disposal. In a joint effort, Dong et al. [113] employed HS-SPME-GC×GC-QTOF-MS in conjunction with chemometrics to meticulously analyze and identify volatile contaminants (VCs) in 57 rPET samples, with the ultimate aim of assessing their authenticity within the geographical recycling regions. An extensive initial analysis of 212 potential VCs sources was conducted, of which 45 compounds were deemed high-priority due to their class IV or V toxicity and potentially dangerous effects on human health. Through stoichiometric analysis, it became apparent that the markers found in rPET samples from the three geographic recovery regions were notably distinct, resulting in an overall accuracy rate of 100% in the initial classification of VCs, with a high degree of cross-validation.

The combination of GC-HRMS technologies and chemometrics presents a novel approach for the detection and analysis of FCMs with the aid of NTS techniques. This approach enables researchers to utilize stoichiometric methods in accordance with the experimental requirements, ultimately facilitating a thorough and comprehensive analysis of compounds found in FCMs. By offering method demonstrations and data support for risk assessment and management of FCMs, this approach offers valuable insights for those engaging in these fields.

## 4. Conclusions and Prospects

The safety of FCMs is a key concern, with their widespread use in everyday life underscoring the need for a comprehensive analysis of the chemicals in them and their migration. Therefore, a comprehensive NTS study is imperative to detect the chemical substances present in FCMs. This should be combined with the careful consideration of environmental factors to ensure full experimentation, providing comprehensive insights into the migration of chemicals in FCMs. Migration experiments must be carried out in detail to verify the migration of these chemicals, taking into account their impact on human health and to determine whether there is any threat posed by the amount of migration.

In selecting the conditions for separating and enriching chemicals found in FCMs made from plastic or paper, it is important to consider both the physical and chemical properties of the corresponding compounds and the complexity of their sources. VOCs and SVOCs are frequently extracted using HS methods and their derivatives, while non-volatile compounds are often removed using solid–liquid extraction methods in combination with field-assisted extraction technology. On this basis, it is also necessary to consider the influence of impurities in plastic/paper-based FCMs. The QuEChERS method is an effective tool for fine tuning the extraction liquid of FCMs to a considerable extent, thus facilitating subsequent detection. In addition, it is worth noting that due to their solid nature, extraction from plastic/paper-based FCMs may pose certain challenges. Therefore, the use of SPE, LLE, SPME, and other enrichment and concentration technologies is essential. In light of the fact that migration experiments have become increasingly common, advances in computer science have led to new developments in this area. By utilizing transfer experiments and direct extraction techniques, experimental data can be more valuable for reference purposes. It is essential to take into consideration both approaches to conducting migration experiments in order to obtain a complete understanding and exploration of compound migration in FCMs.

Given that current targeted screening methods fail to satisfy the detection requirements of chemical compounds within FCM materials, more efficient NTS technologies are imperative. The predominant NTS technology for FCMs is based on HRMS, frequently combined with LC and GC, and involves the use of TOF and Orbitrap instruments, with a further improvement achieved through the inclusion of instruments such as IMS and ASAP. In recent years, the use of NTS technology, mainly LC-HRMS and GC-HRMS, has become more common in plastic/paper-based FCMs, providing a strong basis for the identification and analysis of compounds within these matrices. Nowadays, NTS in FCMs is mainly used in conjunction with LC, GC, and TOF instruments. A wide variety of additives, processing aids, and degradation products in FCMs are tested and analyzed from multiple angles and dimensions, and the potential risks of these substances to human health are assessed through migration experiments. In terms of instrument performance, however, Orbitrap has proven to be more advantageous than TOF in terms of resolution, mass accuracy, sensitivity, linear range, and stability, among other qualities. Therefore, it is worth exploring and investigating how Orbitrap can be used more extensively in NTS research of FCMs. In addition, the integration of computer algorithms, stoichiometric methods, and HRMS represents a novel direction for NTS technologies, revealing significant research potential and warranting further exploration.

Nevertheless, the field of HRMS lacks a mature, publicly available database applicable to NTS research of FCMs. With the promising prospect of this research area, databases should be established to facilitate further research. In the meantime, due to the continuous innovation of pre-processing technology, the integration of computer science and metrology, as well as research attempts in metrology methods, there has been a renewed injection of energy into research on NTS of FCMs. The potential of this research is profound and warrants further investigation.

## Figures and Tables

**Table 1:** Summary of common separation and concentration techniques for plastic/paper-based FCMs.

Type	Extraction Method		Advantage	Disadvantage	Typical Example	Reference
Extraction technology of volatile and semi-volatile compounds	HS		Simplify your pre-treatment and avoid organic solvents, the most simple and commonly used, suitable for GC and ion migration spectrometry	The extraction effect is limited due to the low air pressure and slow mass transfer	Utilizing HS-gas chromatography mass spectrometry (GC-MS) and multivariate statistical analysis, Chen et al. were able to differentiate between primary and recycled polyethylene (PE). Their findings revealed 47 volatile organic compounds (VOCs) within four categories, including aliphatic hydrocarbons and additives. Through the use of orthogonal partial least squares discriminant analysis and non-parametric tests, they successfully identified 16 VOC markers.	[18,19,20]
	Purge and capture technique		Has high sensitivity, good enrichment effect, and high upper boiling point, which is conducive to the detection of trace analytes	Easy to form foam, easy to overload the instrument, time-consuming, may introduce impurities, universal adsorbent selection is difficult	Ibarra et al. used purge and capture technology combined with GC-MS to analyze VOCs and semi-volatile organic compounds (SVOCs) in organic solvent extracts from 12 plastic packaging materials, detecting approximately 100 different compounds.	[21,22,23]
	Headspace solid-phase microextraction technology (HS-SPME)		It can extract VOCs and SVOCs easily and without solvents, while also handling sample collection, concentration, injection, and analysis	Extracting weak volatile components is challenging due to interference from experimental conditions	Parigoridi et al. studied methods to separate plasticizer mixtures from recycled paperboard for food packaging and developed a technique to detect low concentrations of comparable chemicals.	[24,25,26]
	Multi-headspace solid-phase microextraction technique (MHS-SPME)		Trace analysis is more sensitive, matrix effects are gone, accurate quantification results and multiple analytes can be quantified without external calibration.	Headspace saturation needs to be avoided	A study by Salazar et al. analyzed VOCs in polylactic acid particles and identified aldehydes, ethanol, and acetone using MHS-SPME. They also found three specific compounds: acetaldehyde, 2-methyl-2-propanol, and 2,3-pentanedione.	[27,28]
Extraction technology of non-volatile compounds	Solid–liquid extraction combined with field-assisted extraction technology	Microwave-assisted extraction (MAE)	Ensure efficient energy transfer, minimize solvent use, shorten extraction time.	The reaction cycle is lengthy and the operational process is complex	A study conducted by Moreno-Gordaliza et al. analyzed the potential migration of silver ions and microplastics in antibacterial food plastic containers during regular use and microwave heating. The results revealed that in certain scenarios, the migration levels surpassed the acceptable limits of risk.	[29,30]
		Ultrasound-assisted extraction (UAE)	Efficient and simple extraction with low instrument threshold for good results	Sample damage from ultrasonic attenuation makes control and handling difficult.	A review by Peters et al. looked at ways to detect non-intentionally added materials (NIAs) in paper-based FCMs. Methods included UAE for extracting NIAs.	[17,31]
		Accelerated solvent extraction (ASE)	Efficient extraction with minimal solvent and low impact on the body	High equipment requirements	Dorival-Garcia et al. used ASE optimization and LC-HRMS to identify over 100 additives and degradation products in multilayer polymer systems of disposable plastic bags.	[32,33,34]
	The QuEChERS method		FCM impurity purification requirements can be met with this fast, simple, economical, effective, stable, and safe option	The QuEChERS method needs improvement to detect complex FCMs	Diamantidou et al. developed an ultra-high-pressure liquid chromatography(UPLC)-quadruply-time-of-flight (qTOF) mass spectrometry (MS) method for the analysis of NIA migration in FCMs, olive oil, and food simulants in different saturated polyester (PET) bottles.	[35,36,37,38,39]
Enrichment technology	Solid-phase extraction (SPE)		Flexible and diverse, high sensitivity, good reproducibility, can be set sampling, extraction, concentration, sampling in one	The cost is high, the method development is difficult, and it is not suitable for solid samples	Liu et al. established a pipette tip SPE combined with HPLC and photodiode array detector for the detection of atomic absorbents in polyamide (PA) FCMs for migration detection of six atomic absorbents in PA kitchenware.	[40,41,42,43,44]
	Liquid–liquid extraction (LLE)		Simple and fast operation, high selectivity, no special equipment required	Extracting water-soluble compounds from water is tough due to the high organic solvent content and long operation time.	Tsochatzis et al. has developed and refined the salt-out LLE technique to analyze caprolactam and 2,4-di-tert-butylphenol (2,4-DTBP) in water and food simulant samples. The method achieved high accuracy with recovery rates of 87% and 95%, respectively, and a relative standard deviation (RSD) below 12%.	[45,46,47]
	Solid-phase microextraction (SPME)		Easy to operate, efficient and sensitive, can selectively enrich compounds, small sample size	May be affected by interference, selectivity and sensitivity are affected by the sample and solid-phase material	Li et al. utilized oxygenated carbon nanotube cage materials, which were created by oxidizing zeolite imidazole-frame-67, as SPME packages for extracting aromatic amines from FCMs. And they developed a new detection method with gas chromatography-tandem mass spectrometry (GC-MS/MS).	[48,49,50,51,52,53,54,55]
	Liquid-phase microextraction (LPME)		Simple operation, high enrichment efficiency, small extractant dosage, easy to combine with chromatographic system	Extraction solvent, temperature, salt, pH, and stirring affect it easily.	Li et al. designed eight novel low-viscosity hydrophobic eutectic solvents as extractants for eddy-assisted LLME technology to extract and preen rich phthalates from water samples, and determined phthalates content in plastic FCMs using GC-MS.	[56,57,58,59,60]

**Table 2:** Examples of plastic/paper-based FCM migration experiments.

Number	Sample	The Migrants	Food Simulants	Research Content	Reference
1	Spike paper	Photoinitiators such as benzophenone (BP), 2-hydroxybenzophenone (2-HBP)	Tenax^®^, Porapak^®^, and Tylose^®^	The migration behavior of Porapak^®^ was similar to that of Tenax^®^, but Tylose^®^ was slightly lower in the order of 2HBP > BP > 4-hydroxybenzophenone (4-HBP) among all simulants, with the migration amount of 4-HBP significantly lower.	[66]
2	Multilayer PA packaging	ε-caprolactam	Water, 3% acetic acid solution, olive oil	At high temperature and atmospheric pressure, ε-caprolactam migrates more to different simulants than at high pressure, but remains below the permitted specific migration value of 15 mg/kg.	[67]
3	Primary carton packaging	Mineral oil aromatic hydrocarbons (MOAHs)	Modified polyphenoxyethylene	The migration patterns of model compounds are influenced by their volatility and food substrate. The behavior of the most volatile and heaviest compounds is distinctive.	[68]
4	Plastic FCMs	Antioxidant	95% ethanol, water, and 4% acetic acid	Irganox 1010, Irganox 1076, and antioxidant LTDP had the highest detection frequency and concentration	[69]
5	Paper FCMs	Mineral oil hydrocarbons	Tenax^®^	The maximum temperature of Tenax^®^ in paper-based FCMs migration testing should not be higher than 40 °C.	[70]
6	Water	Primary aromatic amines (PAAs)	Water; 3% acetic acid; 10%, 20%, and 50% ethanol	PAAs is most unstable in 3% acetic acid and more stable in 3 mmol/L HCl solution. In ethanol-containing food simulants, most PAAs are stable. Reducing the temperature can improve its stability, and shortening the storage time can improve its recovery rate.	[71]
7	Paper FCMs	Perfluorocarboxylic acid/sulfonic acid (PFCAs/PFSAs), polyfluoroalkyl phosphate (PAPs), and fluoropolyols (FTOHs)	20%, 50% ethanol	Migration of PFCAs and FTOHs to 50% ethanol is higher than migration to real food, while FTOHs do not migrate to 20% ethanol. Children’s estimated dietary exposure to polyfluoroalkyl substances (PFASs) is exceeding the safe threshold and poses a health risk.	[72]
8	Paper and cardboard materials	Photoinitiators, phthalates and plasticizers	50%, 95% ethanol, and Tenax	Tenax was an adequate simulation model for the migration to rice and cereals, but underestimated the migration to infant milk powder, 95% ethanol was a superior simulant for this particular food	[73]
9	Paper and board materials	Per- and polyfluorinated compounds	50% ethanol, 95% ethanol, and Tenax^®^	Tenax^®^-based techniques underestimate the migration of PFASs to food stuffs, particularly for short-carbon-chain PFASs and milk powder.	[74]
10	Contaminated food contact articles	Brominated flame retardants (BFRs)	Water, 3% acetic acid, 10% ethanol and 50% ethanol	HBCD not detected. Phenolic BFRs (tributyl phosphate and tetrabromobisphenol A) migrated in food simulants from nondetected to 73 µg/kg, and in foods from 1 to 23 µg/kg. Phenolic BFRs migrated more into 50% ethanol than aqueous simulants and foods.	[75]
11	Bio-based food packaging material	Brominated flame retardants	96% ethanol	Real samples had low chemical migration in most cases, except for one case. Low percentage suggests low health concern.	[76]

**Table 3:** Examples of NTS analysis of LC-HRMS in FCMs.

Number	Sample	Screened Compound	Instrumental Method	Detection Limit/ Quantification Limit	Detected Quantity	Recovery	Reference
1	FCMs	11 PFAS	UPLC- IMS-QTOF	0.07~3.42/0.20~11.4 μg/kg	3.2~22.3 μg/kg	119 ± 22%	[81]
2	FCM extract	64 small-molecule compounds	UPLC-QTOF-MS	—	0.0001~14.2470 mg/d	—	[93]
3	Liquid food simulator, PA/PE FCM multilayer film	13 PA monomers and oligomers	QTOF-MS	0.6~4.8/1.7~14.5 μg/L	18.1~212.2 ng /mL	78.3%~108.7%	[94]
4	Plastic FCMs for microwave and conventional oven heating	74 kinds of migration compounds	LC-Orbitrap-MS	—	—	—	[95]
5	Juice milk bag, 18 kinds of packaging samples	10 kinds of UV ink photoinitiator and 8 kinds of PAAs	LC-Orbitrap-HRMS	—/0.5~5µg/kg	0.004~658 ng/g	72%~120%	[96]
6	Envelope	22 kinds of plasticizers	UPLC-MS	0.04~10/1.0~50 μg/kg	—	75.6~124.5%	[98]
7	FCM UV varnish	54 kinds of NIAs	UPLC-ion mobility-QTOF-MS	0.01~0.1/— mg/kg	0.01~3.83 mg/kg	—	[99]

**Table 4:** Examples of GC-HRMS NTS analysis in FCMs.

Number	Sample	Screened Compound	Instrumental Method	Detection Limit/ Quantification Limit	Detected Quantity	Recovery	Reference
1	Plastic FCMs for microwave and conventional oven heating	74 kinds of migratory compounds	GC-Orbitrap-MS	—	—	—	[95]
2	Polypropylene-based FCMs	27 kinds of VOCs, SVOCs	APGC-QTOF-MS	—	—	—	[103]
3	FCMs	27 best markers of MOAH	Ambient solid analysis probe (ASAP)/APGC-QTOF-MS	0.01~0.06/0.1~0.3 μg/g	8.62~25.12 mg/kg	—	[104]
4	Paper FCMs	35 kinds of migratory compounds	GC-Orbitrap-MS	—	189~600 μg/kg	—	[105]
5	Polylactic acid and PET blend	15 kinds of VOCs	APGC-QTOF-MS	226~2800/310~8400 µg/kg	—	40.0%~91.3%	[106]
6	Water	12 neutral PFAS substances	HS-SPME-GC-atmospheric pressure-photoionization-HRMS	0.02~0.24/0.08~50 ng/L	—	—	[107]
7	Polypropylene FCMs	45 kinds of VOCs, SVOCs	GC-electron impact (EI)-QMS/GC-EI-TOF-MS	—	5.4%~98.9%	—	[108]

## Data Availability

No new data were created or analyzed in this study. Data sharing is not applicable to this article.

## Abbreviations

ASE	accelerated solvent extraction
APGC	Atmospheric-pressure gas chromatography
ASAP	Ambient solid analysis probe
BP	benzophenone
BFRs	brominated flame retardants
EI	electron impact
FCM	food contact material
GC	gas chromatography
GC × GC	full two-dimensional meteorological chromatography
GC-MS/MS	gas chromatography-tandem mass spectrometry
HRMS	high-resolution mass spectrometry
HS	headspace extraction
HS-SPME	headspace solid-phase microextraction technology
IMS	ion mobility spectroscopy
LC	liquid chromatography
LDPE	low-density polyethylene
LLE	liquid–liquid extraction
LPME	Liquid-phase microextraction
MAE	microwave-assisted extraction
MHS-SPME	multi-headspace solid-phase microextraction technique
MOAH	Mineral oil aromatic hydrocarbons
MS	mass spectrometry
NIAs	non-intentionally added materials
NTS	non-targeted screening
PA	polyamide
PAAs	Primary aromatic amines
PAPs	polyfluoroalkyl phosphate
PE	polyethylene
PET	polyester
PFCAs/PFSAs	Perfluorocarboxylic acid/sulfonic acid
PFAs	Polyfluoroalkyl substances
PTOHs	fluoropolyols
qTOF	quadruply-time-of-flight
rPET	recovered polyethylene terephthalate
RSD	relative standard deviation
SPE	solid-phase extraction
SPME	solid-phase microextraction
SVOCs	semi-volatile organic compounds
TOF	time of flight
UAE	ultrasound-assisted extraction
UPLC	ultra-performance liquid chromatography
VCs	volatile contaminants
VOCs	volatile organic compounds
2,4-DTBP	2,4-di-tert-butylphenol
2-BP	2-hydroxybenzophenone
4-BP	4-hydroxybenzophenone
